# Pharmacogenetics in Pharmaceutical Care—Piloting an Application-Oriented Blended Learning Concept

**DOI:** 10.3390/pharmacy9030152

**Published:** 2021-09-06

**Authors:** Céline K. Stäuble, Chiara Jeiziner, Kurt E. Hersberger, Henriette E. Meyer zu Schwabedissen, Markus L. Lampert

**Affiliations:** 1 Biopharmacy, Department of Pharmaceutical Sciences, University of Basel, 4056 Basel, Switzerland; h.meyerzuschwabedissen@unibas.ch; 2 Pharmaceutical Care, Department of Pharmaceutical Sciences, University of Basel, 4001 Basel, Switzerland; chiara.jeiziner@unibas.ch (C.J.); kurt.hersberger@unibas.ch (K.E.H.); markus.lampert@unibas.ch (M.L.L.); 3 Institute of Hospital Pharmacy, Solothurner Spitäler, 4600 Olten, Switzerland

**Keywords:** pharmacogenetics, pharmacogenetic testing, pharmaceutical care, pharmacy service, blended learning, e-learning, digital training, advanced training

## Abstract

To enable application-oriented training of Swiss pharmacists on pharmacogenetic (PGx) testing, an advanced, digital training program was conceptualized based on the Miller’s Pyramid framework, using a blended learning approach. The PGx advanced training program included an asynchronous self-study online module, synchronous virtual classroom sessions with lectures and workshops, and a follow-up case study for in-depth applied learning including the analysis of the participants’ PGx profile. The evaluation of the training program consisted of (a) an assessment of the participants’ development of knowledge, competencies and attitudes towards PGx testing in the pharmacy setting; (b) a satisfaction survey including; (c) questions about their future plans for implementing a PGx service. Twenty-one pharmacists participated in this pilot program. The evaluation showed: (a) a significant improvement of their PGx knowledge (mean score in the knowledge test 75.3% before to 90.3% after training completion) and a significant increase of their self-perceived competencies in applying PGx counselling; (b) a high level of satisfaction with the training program content and the format (at least 79% expressed high/very high agreement with the statements in the questionnaire); (c) a mixed view on whether participants will implement PGx testing as a pharmacy service (indecisive 8; agreed/completely agreed to implement 7/1; disagreed 3 (*n* = 19)). We consider ongoing education as an important driver for the implementation of PGx in pharmacy practice.

## 1. Introduction

Interindividual differences in response to pharmacotherapies are common and may be caused by various avoidable factors such as drug–drug and food–drug interactions, as well as insufficient adherence, but also by given predispositions such as renal and hepatic deficiency and notably genetics. Hence, today various drug labels point out the impact of genetic predispositions on drug response and in certain cases even strongly recommend genetic testing prior to treatment start [1]. Genetic testing, so called pharmacogenotyping, is used to identify individuals, who may particularly benefit from a certain pharmacotherapy, or who carry an increased risk of therapy failure, adverse drug reactions and even severe toxicities due to their genetic makeup [2]. Currently, multiple guidelines with recommendations for genotype-based drug selection and dosing are available and comprise a variety of actionable drug–gene interactions such as clopidogrel and *CYP2C19* encoding for the enzyme cytochrome P450 2C19, as well as 5-fluorouracil and *DPYD* encoding for the enzyme dihydropyrimidin dehydrogenase [3,4].

However, despite the already accumulating evidence, the adoption of pharmacogenotyping in clinical practice remains modest, which is attributed to multiple barriers such as the restricted reimbursement of pharmacogenetic tests, the limited evidence from prospective clinical trials, as well as a lack of education of health care professionals [5]. In Switzerland, pharmacogenetic (PGx) testing can currently only be initiated by physicians and in the majority of cases even requires a prescription from a specialized pharmacologist to ensure health insurance coverage [6]. It may be discussed that these rather restrictive requirements for the initiation of PGx testing may additionally hamper its implementation in clinical practice. However, currently, the Swiss law on genetic investigations in humans is being revised and a notion to enable pharmacists to initiate PGx testing is under consideration [7]. It may be speculated that the extension of the pharmacist’s competencies in PGx testing may also support further adoption of pharmacogenetics in health care practice.

Nonetheless, as aforementioned limited education of health care professionals remains a relevant barrier in the implementation of PGx testing. In 2018, we conducted a survey among Swiss community (*n* = 238) and hospital (*n* = 134) pharmacists, which showed that about 75% of the participating pharmacists perceived their knowledge of PGx as insufficient to adequately advise their patients. However, the same number of pharmacists considered it their duty to counsel patients in the matter of PGx and additionally expressed willingness to participate in an advanced training course on the said topic [8]. A recent survey among Canadian pharmacists showed comparable results and additionally identified digital training as a highly accepted and favoured way of learning [9].

Digital training is a widely used and studied method in under- and postgraduate pharmacy education, where it was shown to improve the immediate gain of knowledge and enjoys a high acceptance rate amongst participants [10]. Notably, there are various approaches in reported educational programs using digital media, including online modules, online reading materials and online, synchronous, or asynchronous lectures [10]. These also included training programs using a blended learning approach, where synchronous face-to-face classroom teaching and asynchronous online learning were combined [11,12]. The mix of learning environments and the use of digital technologies in blended learning was found to improve the students’ performance in exams [12] and additionally may promote participant engagement. Digital training approaches have also been used in pharmacogenomics education of pharmacists and pharmacy students, where in a recent international survey especially online open access PGx databases were reported to be frequently used [13]. Overall, the adoption of pharmacogenetics into pharmacy and medical university curricula has increased globally in recent years, but seems to be mainly taught on a genetic level without integration into other scientific fields and may therefore lack application-oriented aspects [13]. The Miller’s Pyramid offers a framework for the conceptualization and assessment of application-oriented training for professional services. It defines four levels of performance: (1) knows; (2) knows how; (3) shows how; (4) does [14].

Meanwhile, numerous post-graduate training programs for pharmacists on pharmacogenetics are available, and amongst others are widely offered in the USA and Canada [15,16]. However, to our knowledge, no such in-depth, post-graduate training program is yet available in Switzerland. To fill this educational gap and to properly prepare pharmacists for their anticipated, challenging responsibility in PGx testing, an advanced, digital training program for community and hospital pharmacists was developed. We, a PGx task force group of health care professionals from academia and pharmacy practice, piloted the training program between October 2020 and January 2021, using an application-oriented blended learning concept.

## 2. Materials and Methods

### 2.1. Learning Outcomes

Overall, we aimed to enable the participating pharmacists to identify and address PGx issues arising in their daily work and to support patients and other health care providers accordingly. In particular, the following learning outcomes were defined:

- The pharmacists have knowledge of:
  1. The basics of pharmacogenetics;
  2. The current legal situation in Switzerland concerning genetic testing;
  3. The currently available PGx tests and their reimbursement in Switzerland.
- The pharmacists are able to:
  1. Evaluate the evidence and the implications of PGx testing for clinical practice;
  2. Rate information concerning PGx in the Swiss summary of product characteristics;
  3. Interpret results from PGx tests and incorporate them into a medication review;
  4. Support patients and health care providers likewise with their inquiries about PGx.

### 2.2. Recruitment

For the pilot training program, we aimed to recruit 20 pharmacists interested in clinical pharmacy and interdisciplinary topics, working in hospitals, community pharmacies and other institutions (e.g., authorities). The program was officially announced and open for registration on the website for continuous education of the Department of Pharmaceutical Sciences at the University of Basel. Additionally, we recruited participants via email invitation, who were enrolled at the University of Basel for the advanced certificate studies in clinical pharmacy, or who had subscribed for the email distribution list of the Swiss Association of Public Health Administration and Hospital Pharmacists (GSASA).

### 2.3. Program Design

To allow best possible location- and time-independent learning, we chose a blended learning approach, combining asynchronous, self-study online modules and synchronous, virtual classroom sessions as well as a follow-up with individual case studies for in-depth applied learning. The program content was selected and structured according to the framework of Miller’s Pyramid, which defines four levels for the realization and assessment of a professional service [14] (Table 1). The piloting of the training program was conducted over a period of three months, starting off with an asynchronous, self-study online module using the learning management system ADAM, https://adam.unibas.ch/ (accessed on 18 November 2020) (University of Basel, 2020). This asynchronous, self-study online module was designed to cover the basics of PGx including genetic variation, pharmacologically relevant genotypes and phenotypes, as well as open access sources for PGx information retrieval (Pharmacogenomics Knowledge Base, Dutch Pharmacogenetics Working Group) [17,18]. The participants were able to study independently and self-paced using online content including texts, quizzes, and links to further literature. During this phase, it was also possible to exchange information with participants and instructors via an online discussion board. We anticipated a minimum effort of half a day to complete the asynchronous online module, but the participants were granted time-independent access to the according platform throughout the training program. In preparation for the following synchronous, virtual classroom session at the beginning of the second month, the participants were asked to complete a basic case study covering the contents of the previous asynchronous, online module. The subsequent synchronous, virtual classroom session was held via the video conferencing system Zoom 5.0.4 (Zoom Video Communications, San José, CA, USA). The session contained lectures covering the legal framework for genetic testing in Switzerland, reasonable indications for PGx testing as well as application and interpretation of PGx test results in pharmacy practice. Furthermore, the participants worked in groups of four, in break out rooms of the Zoom session, on three different patient cases using real-life, anonymized pharmacogenetic and health data to solve the cases and come up with recommendations. Following this first virtual classroom training, the participants were given the opportunity to have their personal PGx profile assessed and interpreted using the commercial service Stratipharm^®^ (humatrix AG, Pfungstadt, Germany). Based on their personal and an exemplary PGx profile, the participants were asked to solve a fictional polypharmacy case as a transfer task, writing a report with recommendations for a fictional treating physician. After three months a second synchronous, virtual classroom session was conducted. Herein, the participants were given individual feedback on their transfer task. Furthermore, the program organisers and experts discussed together with the participants the opportunities and challenges for pharmacogenetics in today’s and future pharmacy practice.

### 2.4. Assessments

To assess the participants’ progress in learning as well as their development of competencies and attitudes toward PGx testing in pharmacy practice, we used online self-assessment questionnaires, knowledge tests, and satisfaction surveys (Table 1). In order to start the asynchronous, self-study online module, the participants had to answer a 16-item multiple choice knowledge test on the basics of PGx. This test was developed and reviewed by experienced university educators of our task force group. After completion of the asynchronous self-study online module participants had to answer the knowledge test again, to assess their status and progress concerning PGx basic knowledge. Furthermore, we aimed to evaluate the participants’ development of competencies and attitudes towards PGx testing in pharmacy practice based on their self-perception. Therefore, we applied a self-assessment questionnaire adapted from a recent project on the topic published by Crown and colleagues [15]. In the adapted questionnaire, participants were asked to score their agreement with 13 statements describing their knowledge, competence and attitude towards PGx testing on a five-point Likert scale, before and after attending the complete digital training program. In addition we surveyed the participants’ satisfaction with the training program, using a 67-item questionnaire, which was based on an already available evaluation questionnaire from certified continuous pharmaceutical education courses. The respective questionnaire allowed open-ended and Likert-type responses to assess opinions regarding the content and the realization of the program as well as to collect suggestions on its potential further improvement.

### 2.5. Data Analysis

Participant characteristics and outcomes of the participant satisfaction survey were summarized and described as either means and standard deviations (SD) for scale variables or total counts and percentage for group variables. Due to the small sample size we chose non parametric testing to compare outcomes of the pre- and post-training knowledge test and self-perception questionnaires. For the knowledge test we used the Wilcoxon matched pairs test. However, as the method of data collection in the self-perception questionnaires did not allow data pairing, pre- and post-training outcomes were compared using the Mann–Whitney test. GraphPad Prism Version 5.01 was applied for all statistical analyses.

## 3. Results

### 3.1. Participant Characteristics

The group of 21 participants (women *n* = 14, 66.7%; mean age = 38 years, SD 8.9), consisted of pharmacists with an average of 12.5 (SD 8.2) years of practical experience and a majority holding a postgraduate degree in community or hospital pharmacy (*n* = 15; 71.4%). Participants predominantly had a hospital pharmacy background (*n* = 12; 57.1%) and were almost exclusively from the German speaking part of Switzerland (*n* = 20; 95.2%) (Table 2).

### 3.2. Participant Development of Knowledge, Attitude, and Competence

Prior to the asynchronous, self-study online module all participants (*n* = 21) answered the multiple choice PGx knowledge test and scored on average 75.3% (SD 8.9). The mean score of the PGx knowledge test participants took after the asynchronous, self-study online module resulted in 90.3% (SD 6.0), which is a mean difference of 15% (*p* < 0.001).

The survey of self-perception of knowledge, attitude and competence was collected from all 21 participants prior to the start of the training program, whereas after completion of the full training program only 20 participants answered the follow-up survey. After finishing the training program, participants on average rather agreed with statements expressing knowledge in the field of pharmacogenetics, which is a significantly increased agreement compared to their rating of the same statements prior to the course (Table 3). When asked to rate statements about their attitude towards pharmacogenetics in pharmacy practice, participants rather agreed with them before and after the training program. Statements on competencies to apply pharmacogenetics in practice were perceived as neutral to rather agreeing before the training. After completion of the program participants showed significantly enhanced agreement with the same statements about competencies (Table 4).

### 3.3. Participant Satisfaction

The participants did not consistently answer all questions of the satisfaction questionnaire, which is why the number of answers may differ from the total number of participants. Overall participants expressed their satisfaction with the training program, by mainly strongly agreeing to recommend the course to a colleague (79% (15), *n* = 19) and mainly rating the complete course as excellent (79% (15), *n* = 19). The asynchronous, self-study online module was generally rated as good or excellent (29/67% (6/14), *n* = 21) and participants largely agreed or strongly agreed with the user-friendliness of the learning management system used (38/43% (8/9), *n* = 21) as well as with the desire to attend further trainings with similar online modules (48/38% (10/8), *n* = 21). When asked about the usefulness of the assessment of their individual PGx profile, all participants agreed or completely agreed with it having an additional educative effect (15/85% (3/17), *n* = 20). We further asked about their intentions to implement PGx testing as a service in their pharmacy. A majority expressed to be still indecisive about implementing PGx testing (42% (8), *n* = 19) and others agreed or completely agreed to implement it (36/5% (7/1), *n* = 19). The remainder disagreed with implementing a PGx service in their pharmacy (16% (3), *n* = 19). To explain their reluctance towards introducing a PGx service, participants mentioned lack of time or support from superiors, the current legal requirements regarding the initiation of genetic testing in Switzerland, and the lack of coverage of the service by health insurers. For the overall program, a potential for improvement was especially noted for the extent and time required in the asynchronous learning sequences (self-study online module and transfer task) as well as for the limited opportunities for individual exchange between the participants.

## 4. Discussion

We conceptualized and piloted an extensive multi-day and application-oriented advanced, digital training program on PGx testing as a pharmacy service, which to our knowledge is the first of its kind reported for Switzerland.

The 21 participants showed good knowledge on the fundamentals of pharmacogenetics already prior to the course, measured by the average of correctly answered questions in the initial knowledge test of over 75%. However, after completion of the asynchronous self-study online module, participants further increased their average scores to 90%, which is a significant improvement (*p* < 0.001), indicating a gain of short time knowledge. Additionally, the according standard deviation was reduced by almost one third (±8.9% vs. ±6.0%), when comparing the before and after test results, which indicates a reduction in scattering of test results and therefore a potentially more balanced level of basic PGx knowledge among participants after completing the self-study online module. This asynchronous self-study online module was indeed designed to improve the participants’ knowledge on PGx fundamentals but should also allow for an individual and self-paced familiarization with the topic to bring all participants to a similar level of knowledge. In terms of personal attitude, participating pharmacists were from the beginning convinced about the importance of PGx in pharmacy practice and further about their significant role as pharmacists in PGx testing. The already apparent good PGx basic knowledge and favorable attitude towards PGx testing in pharmacy practice prior to the program, does not necessarily reflect the general Swiss pharmacist community, but is probably due to a selection bias. Paid program participation was open to all pharmacists and registration was on a voluntary basis, which is why we expected to attract and recruit individuals with an already positive attitude towards pharmacogenetics in pharmacy practice and a general interest in the topic. It may be discussed that the already initially present motivation and positive attitude has additionally positively influenced the participants’ learning outcomes.

The average participant could be described as a rather experienced pharmacist, with an average age of over 38 years, experience of over 12 years in pharmacy practice and predominantly holding a postgraduate pharmacy degree. As the course was held in German, we anticipated to mainly attract pharmacists from the German-speaking part of Switzerland, which may indeed limit our findings to this region. However, German is the main language of over 60% of the Swiss population [19].

Participants not only showed an improvement of basic PGx knowledge, but also demonstrated a significant development in self-perceived knowledge and competencies. After completion of the program, the participating pharmacists on average agreed or fully agreed with statements regarding their PGx knowledge and their competencies in applying it in pharmacy practice. Notably, participants perceived their ability to counsel and share information on PGx with a patient as significantly improved. All our reported findings are in line and comparable with other published PGx continuing education programs for pharmacists conducted in Canada and the United States [15,20,21]. In comparison with these previous programs, it is worth mentioning, that in our program we offered the participants an analysis of their personal PGx profile, which may have allowed them to experience the handling and use of PGx data in a more intuitive and therefore sustainable way. Indeed, participants perceived this opportunity as an additional educational benefit. In a recent study it was shown, that after providing medical doctors with their individual PGx profile, their attitude towards PGx testing and awareness of its potential impact on drug therapy significantly changed. The involved medical doctors were more positive about PGx testing and its utility [22]. Another method to enhance sustainable learning, might include the use of simulated patient interactions. Simulation-based clinical pharmacy training has been found to beneficially impact learning in students and postgraduate pharmacists [23]. However, in the herein presented training program we have used non-simulated, theoretically discussed patient cases. To further address patient interaction in PGx counselling simulation-based training might offer a suitable learning method.

Furthermore, our program was conducted exclusively digitally. Initially, only the introductory module on PGx basics was planned as a digital training. However, due to the ongoing COVID19 pandemic at that time, the complete course was later converted into a digital training with alternating sequences of asynchronous and synchronous learning, so called blended learning. The participants’ overall satisfaction with the execution of the program is reflected in their exceedingly positive rating in the final satisfaction survey. Nonetheless, the participants also mentioned a specific drawback of the digital program. They perceived the opportunities for individual exchange between the participating peers as limited. In the case of a further completely digitally conducted program, we would like to specifically ensure that the participants have sufficient opportunities for personal exchange. This may, for example, be enhanced with a kick-off, face-to-face video conference, where participants have the chance to introduce themselves and learn about other participants’ and the overall program goals, to create more of a team spirit.

Connecting and learning from each other’s experiences may play an important role in the further process of implementing a new pharmacy service. When we asked the participating pharmacists after completing the program about their intentions to implement PGx testing as a service in their pharmacy practice, feedback was mixed. Potential barriers to an implementation of PGx testing were defined as a lack of time or support from superiors, the current legal requirements regarding the initiation of genetic testing in Switzerland, and the lack of coverage of the service by health insurers. To help participants gain confidence and offer support with implementation, we initiated a peer group for pharmacists interested in offering PGx services to share their experiences with peers and adressing further challenges met in daily practice (e.g., patient couselling and education, insurance, data protection).

## 5. Conclusions

The pilot study on our advanced, digital training program showed measurable improvement of both knowledge and competencies in applying pharmacgenetic testing in pharmaceutical care. Nonetheless, we consider ongoing education, for example within our peer group, as an important driver for the implementation of PGx testing in community and hospital pharmacies. For the future, we hope to also include interested medical doctors in our educational program and in our peer group, to further enhance the undoubtedly necessary interprofessional collaboration in PGx testing to improve patient outcomes [24].

## Figures and Tables

**Table 1:** PGx training program components and assessments charted to Miller’s Pyramid framework.

Miller’s Pyramid Level	Description	Training Component	Assessment
Level 1: Knows	Knowledge of facts	Asynchronous self-study online module	Knowledge test
Level 2: Knows How	Competences in application	Synchronous virtual classroom (part 1)	Self-assessment questionnaire
Level 3: Shows How	Performance and demonstration of learning	Asynchronous case study with individual PGx profile and synchronous virtual classroom (part 2)	Self-assessment questionnaire and individual feedback
Level 4: Does	Action and integration into practice	Peer group for experience sharing	not available

**Table 2:** Participant characteristics.

Characteristic	Category	Mean (SD) or Number (%)
Age	-	38.1 (8.9) range: 26–57
Gender	Women	14 (66.7)
	Men	7 (33.3)
Years in practice	-	12.5 (8.2) range: 1–32
Practice setting	Community pharmacy	7 (33.3)
	Hospital pharmacy	12 (57.1)
	Other ^1^	2 (9.5)
Postgraduate degree	-	15 (71.4)
Career level	Senior pharmacist	6 (28.6)
	Employed pharmacist	12 (57.1)
	Not specified	3 (14.3)
Percentage employment	-	82.9 (18.7) range: 40–100
Place of work in Switzerland (CH)	German speaking CH	20 (95.2)
	Italian speaking CH	1 (4.8)
	French speaking CH	0 (0)

^1^ Health authorities, Academia.

**Table 3:** Self-perception of knowledge.

Item	Pre-Training Mean (SD) *n* = 21	Post-Training Mean (SD) *n* = 20	*p*-Value (Mann–Whitney Test)
I am sufficiently informed about the availability of genetic testing.	1.8 (0.9)	3.9 (0.8)	<0.001
I am adequately informed about the use of pharmacogenetics in the context of drug selection.	2.1 (0.9)	4.4 (0.6)	<0.001
I am adequately informed about the use of pharmacogenetics in the context of drug dosing.	2.0 (0.7)	4.2 (0.5)	<0.001
I feel comfortable using my current knowledge of pharmacogenetics to recommend medications.	2.0 (1.0)	3.9 (0.4)	<0.001
I feel comfortable using my current knowledge of pharmacogenetics to recommend drug dosages.	1.9 (0.9)	3.8 (0.6)	<0.001

Response Scale: 1 = Do not agree at all; 2 = Rather do not agree; 3 = Neutral; 4 = Rather agree; 5 = Fully agree.

**Table 4:** Attitude and self-perception of competence.

**Item**	**Pre-Training** **Mean (SD) *n* = 21**	**Post-Training** **Mean (SD) *n* = 20**	***p*-Value** **(Mann–Whitney Test)**
Pharmacogenetics will be an important component of pharmacy practice in the future.	4.1 (0.7)	4.2 (0.7)	0.989
As a pharmacist, I am well positioned to interpret information from pharmacogenetics testing for my patients.	4.0 (0.7)	4.2 (0.6)	0.204
Pharmacogenetics is relevant to my clinical practice.	3.9 (0.9)	3.8 (0.9)	0.847
I can identify drugs for which pharmacogenetic testing is an option.	3.1 (1.0)	4.4 (0.5)	<0.001
I am able to accurately apply pharmacogenetic concepts to select medications for my patients.	2.2 (0.9)	3.9 (0.4)	<0.001
I am able to accurately apply pharmacogenetic concepts to determine dosages for my patients.	2.0 (0.8)	3.8 (0.5)	<0.001
I can share information with my patients about how pharmacogenetics can affect the efficacy of their medications.	3.4 (1.0)	4.5 (0.5)	<0.001
I can share information with my patients about how pharmacogenetics can affect the safety of their medications.	3.3 (1.0)	4.5 (0.5)	<0.001

Response Scale: 1 = Do not agree at all; 2 = Rather do not agree; 3 = Neutral; 4 = Rather agree; 5 = Fully agree.

## Data Availability

The data presented in this study are available on request from the corresponding author. Data were collected in German and are not publicly available for privacy reasons.
